# Bathyal octopus, *Muusoctopus leioderma*, living in a world of acid: First recordings of routine metabolic rate and critical oxygen partial pressures of a deep water species under elevated *p*CO_2_

**DOI:** 10.3389/fphys.2022.1039401

**Published:** 2022-12-01

**Authors:** Lloyd A. Trueblood, Kirt Onthank, Noah Bos, Lucas Buller, Arianna Coast, Michael Covrig, Ethan Edwards, Stefano Fratianni, Matthew Gano, Nathaniel Iwakoshi, Eden Kim, Kyle Moss, Chantel Personius, Stephanie Reynoso, Cheyne Springbett

**Affiliations:** ^1^ Biology Department, La Sierra University, Riverside, CA, United States; ^2^ Biology Department, Walla Walla University, College Place, WA, United States

**Keywords:** routine metabolic rate, aerobic metabolism, ocean acidfication, muusoctopus, critical oxygen partial pressure, bathyal, oxygen supply capacity

## Abstract

Elevated atmospheric CO_2_ as a result of human activity is dissolving into the world’s oceans, driving a drop in pH, and making them more acidic. Here we present the first data on the impacts of ocean acidification on a bathyal species of octopus *Muusoctopus leioderma*. A recent discovery of a shallow living population in the Salish Sea, Washington United States allowed collection *via* SCUBA and maintenance in the lab. We exposed individual *Muusoctopus leioderma* to elevated CO_2_ pressure (*p*CO_2_) for 1 day and 7 days, measuring their routine metabolic rate (RMR), critical partial pressure (*P*_*crit*_), and oxygen supply capacity (*α*). At the time of this writing, we believe this is the first aerobic metabolic data recorded for a member of *Muusoctopus.* Our results showed that there was no change in either RMR, *P*_*crit*_ or α at 1800 µatm compared to the 1,000 µatm of the habitat where this population was collected. The ability to maintain aerobic physiology at these relatively high levels is discussed and considered against phylogeny and life history.

## Introduction

Presently atmospheric CO_2_ levels are over 400 ppm (Dunn et al., 2020) well above pre industrial levels of approximately 275 ppm (Macfarling et al., 2006). The world’s oceans absorb as much as one-third of annual anthropogenic CO_2_ (Doney et al., 2009), causing an increase in oceanic partial pressure of CO_2_ (*p*CO_2_). The increased *p*CO_2_ drives a decrease in pH, causing oceanic pH to decline from its pre-industrial revolution level of 8.2 to a current average below 8.1, in a process termed ocean acidification (OA) (Caldeira and Wickett, 2003).

Initial studies of the impact of OA on marine organisms focused largely on challenges faced by calcifying organisms (Fabry et al., 2008). However more recently there has been a number of studies that examine how OA impacts the physiology of an array of organisms. Changes in pH have been shown to impact respiratory physiology (Miller, 1985; Bridges, 1995; Widdicombe and Spicer, 2008; Seibel, 2016). Negative impacts to respiratory physiology make it more difficult to obtain oxygen from the environment and may limit aerobic energy production. This has been shown in crab, squid, fish, and sipunculids (Portner and Zielinski, 1998; Langenbuch and Portner, 2002; Metzger et al., 2007; Rosa and Seibel, 2008; Munday et al., 2009; Walther et al., 2009).

Studies within cephalopods have shown various, and sometimes conflicting, responses to environmental hypercapnia and the resulting low pH. At environmentally relevant ranges of 700–1700 μatm adult Cuttlefish *Sepia officinalis* show no change in aerobic metabolic rate (Gutowska et al., 2008) whereas embryonic *S. officinalis* showed an increase in routine metabolism (Rosa et al., 2013). In squid, the vertically migrating epipelagic squid *Dosidicus gigas* showed conflicting responses with some showing metabolic depression (Rosa and Seibel, 2008) and others showing no effect (Birk et al., 2018). *Sepioteuthis lessoniana* had no aerobic metabolic response below 2000 µatm *p*CO_2_ (Hu et al., 2014). Alternatively the benthic, inter- and sub-tidal octopus *Octopus rubescens* had a short term (1 day) increase in routine metabolic rate (RMR) which then returned to pre-exposure levels within 1 week. Additionally, critical oxygen partial pressure (*P*_*crit*_) was significantly higher after long term exposure to elevated *p*CO_2_ (Onthank et al., 2021).

In studies where *p*CO^2^ is pushed above 2,000 µatm there is a continued variation in response. Cuttlefish increase calcification of their cuttlebone (Gutowska et al., 2008; Gutowska et al., 2010), whereas squid statoliths are malformed and more porous (Kaplan et al., 2013). Above 4,000 µatm squid continue to show a decrease in aerobic metabolic rate (Hu et al., 2014).

**FIGURE 1:**
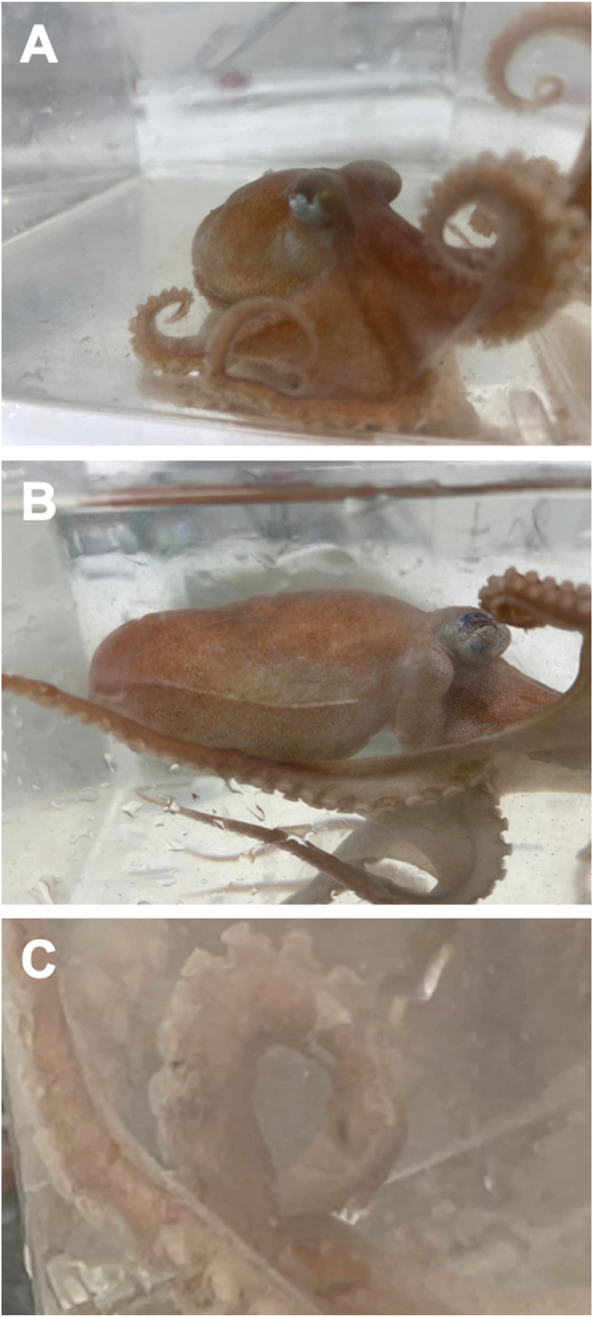
*Muusoctopus leioderma* collected at Burrows Bay, Anacortes Washington, United States **(A)**. Note the well-developed ridge of skin on the periphery mantle **(B)** and hectocotylized arm on third armright **(C)**.

The majority of studies which examine the impact of OA on cephalopods have focused primarily on cuttlefish and squid. As of this writing there has only been one other study we are aware of that explored how OA affects octopus and no studies on bathyal occurring species of octopus. Here, we examine the impact of OA on the Smoothskin octopus *Muusoctopus leioderma* (Berry, 1911). Like all other members of Family Enteroctopodidae, *M. leioderma* is a deep living species. It can be found in the Northern Pacific from the Sea of Okhotsk off Siberia to California and are reported to live on muddy or silty bottoms at meso and bathyal depths ranging from 250 to 1400 m (Conners et al., 2016). This range overlaps with an oxygen minimum zone that has both low oxygen levels as well as *p*CO_2_ levels in excess of 2000 µatm (Kamykowski and Zentara, 1990; Paulmier et al., 2011). The greatest frequency of occurrence for *M. leioderma* has been reported between 450–650 m (Conners et al., 2016). However, it has previously been reported as shallow as 70 m (Hochberg, 1998). Recently a population has been found at depths reachable by SCUBA (10–15 m) in Burrows Bay, Skagit County, Washington, United States, the shallowest record for any individuals in the genus *Muusoctopus*, a major deep water octopus genus with 28 recognized species. Morphological and genetic data were used to confirm the species identity of this population as *Muusoctopus leioderma* (Onthank unpublished data) (Figure 1)*.*

As part of a marine environmental physiology course at the Rosario Beach Marine Lab (RBML) *Muusoctopus leioderma* were collected and held at 1,000 or 1800 µatm for 1 day and 7 days. Routine metabolic rate, critical partial pressure (*P_crit_*) and oxygen supply capacity (*α*) were recorded for each treatment. The Salish Sea, where the RBML is located, is a unique location for OA studies as CO_2_-rich water from the California Undercurrent wells up into this shallow basin producing persistent hypercapnic conditions (Murray et al., 2015). The *p*CO_2_ regularly reaches 1,000 µatm (Onthank et al., 2021). Habitats such as the Salish Sea will experience accentuated acidification due to local hypoxia and eutrophication (Cai et al., 2011; Melzner et al., 2013). The goal of this study was to examine the impacts of short-term and prolonged exposure to hypercapnia on aerobic metabolism of a bathyal associated species of benthic octopus. The population of *M. leioderma* we used for this study was found at depths, and locations near populations of *O. rubescens*, and likely experiences similar environmental conditions. Because of this we hypothesized that *M. leioderma* would show a similar response to elevated *p*CO_2_ as *O. rubescens* having a short term increase in RMR after 24h exposure, and a return to pre-exposure RMR and elevated *P*_*crit*_ with a prolonged 7-day exposure to hypercapnic conditions. This is the first publication of aerobic metabolic rate for any species in the genus *Muusoctopus.* This is also the first study of effects of ocean acidification in any deep water living species of octopus.

## Methods

### Field seawater pH measurement

This research was carried out at the Rosario Beach Marine Laboratory in Anacortes, Washington United States. Water samples were taken at the octopus collection site in Burrows Bay. Samples were taken at depth where the octopus were collected *via* SCUBA. A 50 ml high-density polyethylene sample container filled with air was opened at depth and filled with a water sample, excluding all air bubbles. A screw top lid was used to cap the sample. All water samples were immediately transported to the RBML where pH on the total scale (pH_T_) was measured using the *m*-cresol purple spectrophotometric method (Dickson et al., 2007) within 3 h. Alkalinity was determined by open-cell titration (Dickson et al., 2007), and alkalinity values were calculated from titration data using the at() function in the “seacarb” package version 3.2.14 in R (Gattuso et al., 2015). The resulting measured alkalinity and pH were used to calculate the *p*CO_2_ using the carb() function in the “seacarb” package in R. Samples from the collection site had *p*CO_2_ ∼1,000 µatm.

### Octopus collection

**FIGURE 2:**
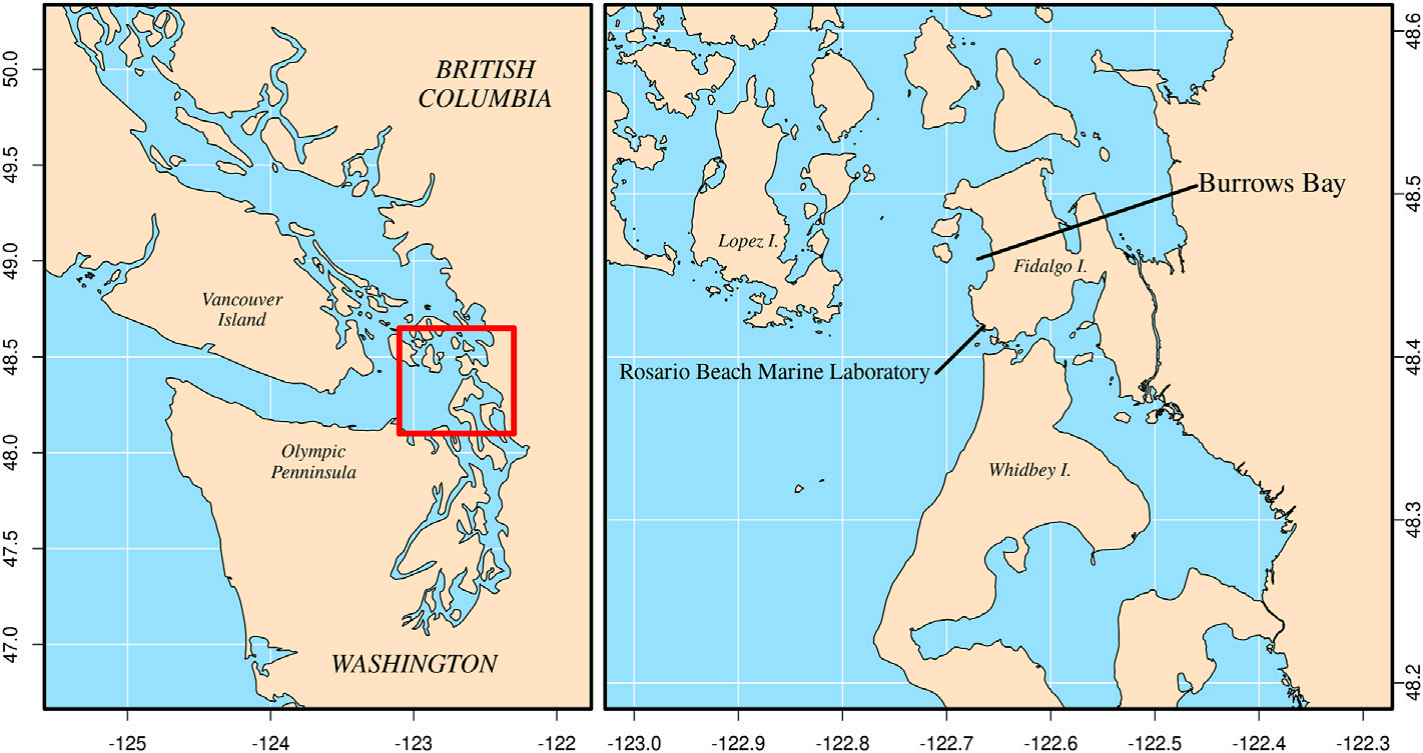
Location of Burrows Bay, Anacortes, Washington, United States where *Muusoctopus leioderma* were collected for this study.

Seventeen *Muusoctopus leioderma* (mass = 2.5–70.0 g) were collected in June through August of 2021 from Burrows Bay, Skagit County, Washington State, United States by SCUBA at depths of 10–20 m (Figure 2). Octopuses were found on the sediment bottom during night dives. Individuals were placed in plastic resealable bags for transport to RMBL. At RMBL octopuses were placed in holding aquaria with sediment that had previously been collected from Burrows Bay. The holding aquaria were supplied with unmodified seawater directly from the lab seawater system which uses seawater pumped from Rosario Bay.

### pCO_2_ level selection

The Salish Sea has persistent hypercapnic conditions that reach 1,000 µatm CO_2_ (Murray et al., 2015) and in the future may increase by an additional 800 µatm (Barry et al., 2010; Cai et al., 2011; Melzner et al., 2013; Bianucci et al., 2018). At the time of octopus collection, water samples from Burrows Bay were approximately 1,000 µatm, which is consistent with values recorded previously by Onthank et al. (2021) at surface and 15 m depth. The selected *p*CO_2_ range allowed us to examine whether the Burrows Bay population that regularly experiences 1,000 µatm CO_2_ would be impacted by the predicted increased CO_2_ levels resulting in 1800 µatm CO_2_ locally.

### Hypercapnia exposure

After being held in aquaria for at least 1 day, octopuses were transferred to treatment tanks as previously described (Culler-Juarez and Onthank, 2021). Each tank (113.5 L) was made using an insulated cooler with an overhead window in the lid to allow observation of octopus. The tank system included an Active Aqua AACH10HP chiller to maintain temperature, and a venturi injector to keep tank water oxygenated. Each tank had a slow constant water exchange from the lab sea water system which flowed fresh seawater into the tank at ∼100 ml min^−1^ and drained from an overflow port. This prevented building up waste in each tank without having to perform large, full-tank water changes. Temperature and *p*CO_2_ of each tank was controlled with a pH-stat system (https://open-acidification.github.io/) which received temperature input from a PT-100 temperature probe and pH input from a single junction glass pH electrode. The pH of aquaria was measured daily using the *m-*cresol purple spectrographic method (Dickson et al., 2007), and that measured aquarium pH used to perform a one-point calibration of the pH electrode. Temperature probes were calibrated using a 0.1°C resolution alcohol thermometer. To modify *p*CO_2_ pure gaseous CO_2_ was slowly bubbled into the tank by a solenoid controlled *via* the Open Acidification Tank Controller. Temperature was controlled by powering on/off a chiller controlled by the same Tank Controller.

In addition to pH measurements, the total alkalinity (A_T_) was measured weekly using a modified open cell titration based on Dickson et al. (2007). Off gassing time was increased from 6 to 10 min with vigorous stirring *via* a stir bar and motor. Titrations were verified against certified reference material (CRM, supplied by Andrew Dickson, Scripps Institution of Oceanography, San Diego, CA, United States). The alkalinity values were calculated from titration data using the “seacarb” package in R (Gattuso et al., 2015). The measured alkalinity and desired *p*CO_2_ of each tank were used to calculate pH setpoints and tank pH set points were updated weekly. The pH was calculated from raw spectrographic data using the specpH(), function in the OTools package in R (https://github.com/KirtOnthank/OTools), alkalinity and PCO_2_ were determined using the at() function and carb function respectively in the seacarb package in R (https://CRAN.R-project.org/package=seacarb).

After at least 1 day acclimating in holding aquaria, octopus were transferred to treatment tanks. One octopus was placed in a treatment tank and held at either 1,000 µatm or 1800 µatm for 1 day and then its RMR and *P*_*cri*t_ were measured (methods described below) and it was then returned to the same treatment tank and held for additional 6 days, for a total of 7 days exposure to its treatment *p*CO_2_. Routine metabolic rate and *P*_*crit*_ were then measured again after the 7th day of exposure. During treatment feeding was done by placing purple shore crabs in the treatment tank after the first 1 day RMR data collection and removed 1 day prior to RMR and *P*_*crit*_ measurements on day 7.

### Routine metabolic rate measurement

Routine metabolic rate were measured after fasting for 24 h. Octopus were placed in 1 L or 120 ml as body size required, flow through water-jacketed respirometers. Experimental temperatures were between 13°C and 14°C. The same type of pH-stat system that was used in the treatment tanks was used to adjust the pH of the seawater for the flow through respirometry. PyroScience Firesting or Presense O_2_ flow-through optode cells and robust temperature probes were placed on the incurrent and excurrent channel of each respirometer. A peristaltic pump was used to cycle water through the system. Flow rates for each respirometer were measured at the start and end of each respirometry run by measuring output of water mL for 1 minute. Flow rates for 1 L respirometers had a mean rate of 30 ml min^-1^ and 120 ml respirometers had a mean flow rate of 7 ml min^-1^. Octopuses were placed in the respirometers for 24 h and aerobic metabolic rates were measured throughout; during analysis, the first 3 hours of each RMR run was trimmed out to account for any handling stress on metabolism. Because the octopus were able to spontaneously move in their respirometer, though they typically did not, we termed our metabolic rates routine metabolic rate instead of standard metabolic rates. After RMR and *P*_*crit*_ experiments were completed, the octopuses were removed and oxygen consumption was measured in the empty respirometer to determine back-ground respiration. After the first eight runs, background respiration was consistently 5% or less of octopus respiration, and was therefore not recorded for the remainder of the study. After background respiration was measured, inflow and outflow optodes were connected immediately in series to evaluate drift. Throughout all experiments no drift was detectable. RMR was calculated from raw oxygen data using the resp.open() function in the OTools package in R (https://github.com/KirtOnthank/OTools). In brief, this function calculates RMR by subtracting the oxygen concentration of the outflow water from the oxygen concentration of the inflow water, multiplying this by the flow rate, and dividing by the mass of the octopus.

### Oxygen supply capacity and critical oxygen pressure measurement

Following 24 h RMR measurements, the respirometer was closed by connecting the inflow of the respirometer to the outflow. Oxygen concentration in the respirometer was allowed to fall to at least 50 µmol O_2_ l^−1^. Oxygen supply capacity (*α*) and *P*_*crit*_ was determined from aerobic metabolic rate (R) as function of oxygen partial pressure (P_O2_). We used the calc_alpha function to determine *α* and determined the *P*_*crit*_ *using the* α-method described in Seibel et al. (2021) using the calc_pcrit() function in the “respirometry” package in R (https://CRAN.R-project.org/package=respirometry).

### Statistical analysis

The effects of *p*CO_2_ exposure on the log of RMR, *α*, and *P_crit_* were examined using repeated-measures linear mixed effect models with log of mass, *p*CO_2_ and duration in treatment included as fixed factors and octopus ID as a random factor using the lme() function in the “nlme” package in R (Pinheiro et al., 2022). Estimated marginal means (covariate-corrected means, in this case, mass) were determined for each *p*CO_2_ category using the emmeans package in R (Lenth, 2018).

### Data availability

All of the raw datasets, data ID files, and R scripts underlying all statistical analysis and figures presented in this study can be found at the Zenodo online repository: https://doi.org/10.5281/zenodo.7058934.

## Results

Control treatments had a measured *p*CO_2_ of 1,083 ± 48 µatm; high CO_2_ treatments had a measured *p*CO_2_ of 1767 ± 94. (Table 1).

**TABLE 1:** Carbonate system parameters of control and experimental tanks. The pH was measured daily for each of the four control and four experimental tanks, (*n* = 29 per tank), Alkalinity and salinity were measured weekly including day one and the last day of the experiments (*n* = 6 per tank). Means are presented with ± as standard error.

Treatment	pCO_2_ (µatm)	pH	Alkalinity (µmol kg^−1^)	Salinity (PSU)
Control	1,083 ± 48	7.621 ± 0.019	2068 ± 7	30 ± 0.4
Elevated CO_2_	1767 ± 94	7.422 ± 0.022	2066 ± 4	30.1 ± 0.4

**FIGURE 3:**
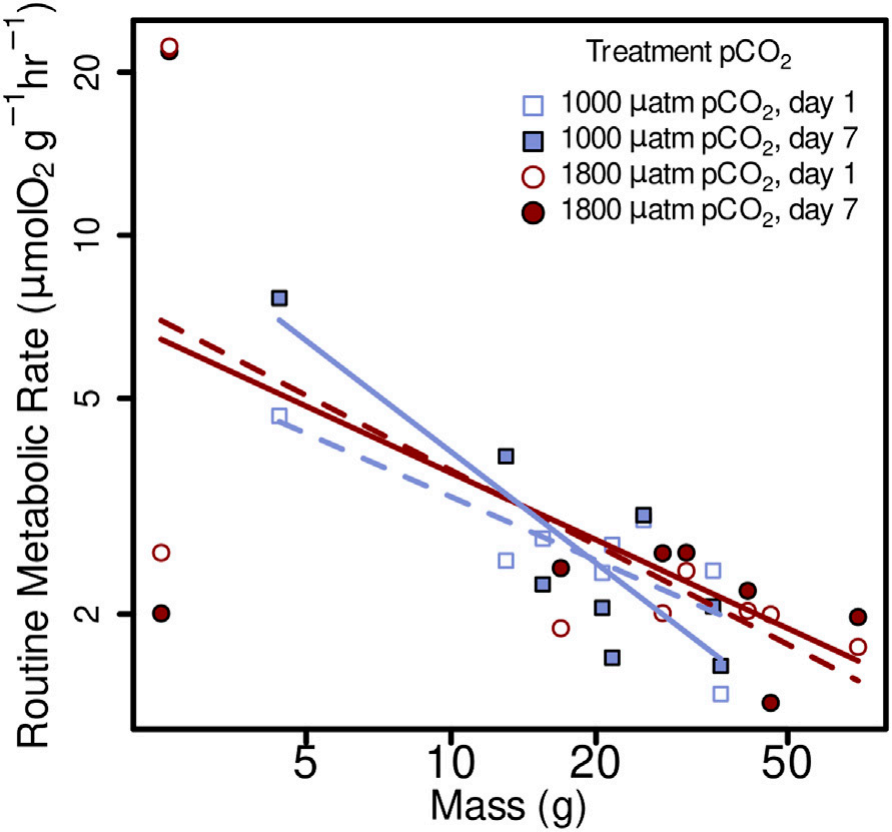
Routine metabolic rate (RMR) from *Muusoctopus leioderma* in Burrows Bay, Anacortes Washington held for 1 day (open symbols) and 7 days (closed symbols) at 1,000 µatm (purple squares) or 1800 µatm (maroon circles) *p*CO_2_ expressed as a function of body mass (M). Routine metabolic rate was significantly affected by body mass (M), decreasing mass specific RMR with increased body mass in all four treatments as follows: 1,000 µatm 1 day RMR = 8.06 M^−0.39^, 1,000 µatm 7 day RMR = 19.17 M^−0.68^, 1800 µatm 1 day RMR = 10.61 M^−0.46^, and 1800 7 day RMR = 9.36 M^−0.41^. Note that axes are plotted on a log scale.

There was a significant effect of mass (linear mixed-effects model, *x* ^*2*^ = 5.84, df = 1, *p* = 0.01565), but not of *p*CO_2_ (linear mixed-effects model, *x* ^*2*^ = 0.19, df = 1, *p* = 0.6621) nor day of measurement (linear mixed-effects model, *x* ^*2*^ = 2.18, df = 1, *p* = 0.13939) on the RMR of *Muusoctopus leioderma*. Routine metabolic rate for 1 day exposure showed an estimated marginal mean (EMM) at 1,000 µatm *p*CO_2_ = 2.60 µmol O_2_ g^−1^ hr^−1^ and at 1800 µatm *p*CO_2_ = 2.79 µmol O_2_ g^−1^ hr^−1^ and for 7 days exposure RMR 1000 µatm *p*CO_2_ = 2.64 µmol O_2_ g^−1^ hr^−1^ and at 1800 µatm *p*CO_2_ = 2.84 µmol O_2_ g^−1^ hr^−1^ (Table 2; Figure 3).

**TABLE 2:** Estimated marginal means for routine metabolic rate of *Muusoctopus leioderma* held at either 1,000 or 1800 µatm *p*CO_2_ for 1 day and 7 days. These values are corrected for mass of 18.3 g, the value that corresponds to the mean of the logged masses from the linear mixed effects analysis. For all treatments *n* = 8.

pCO_2_ (µatm)	day	RMR (µmol O_2_ g^−1^ hr^−1^)	RMR 95% CI
1,000	1	2.60	1.75–3.87
1800	1	2.79	1.88–4.16
1,000	7	2.64	1.77–3.93
1800	7	2.84	1.91–4.23

Routine metabolic rate was significantly affected by body mass (M), decreasing mass specific RMR with increased body mass in all four treatments as follows: 1,000 µatm 1 day RMR = 8.06 M^−0,39^, 1,000 µatm 7 day RMR = 19.17 M^−0.68^, 1800 µatm 1 day RMR = 10.61 M^−0.46^, and 1800 7 day RMR = 9.36 M^−0.41^ (Figure 3; linear mixed-effects model, *x* ^2^ = 5.84, *p* = 0.0157).

**FIGURE 4:**
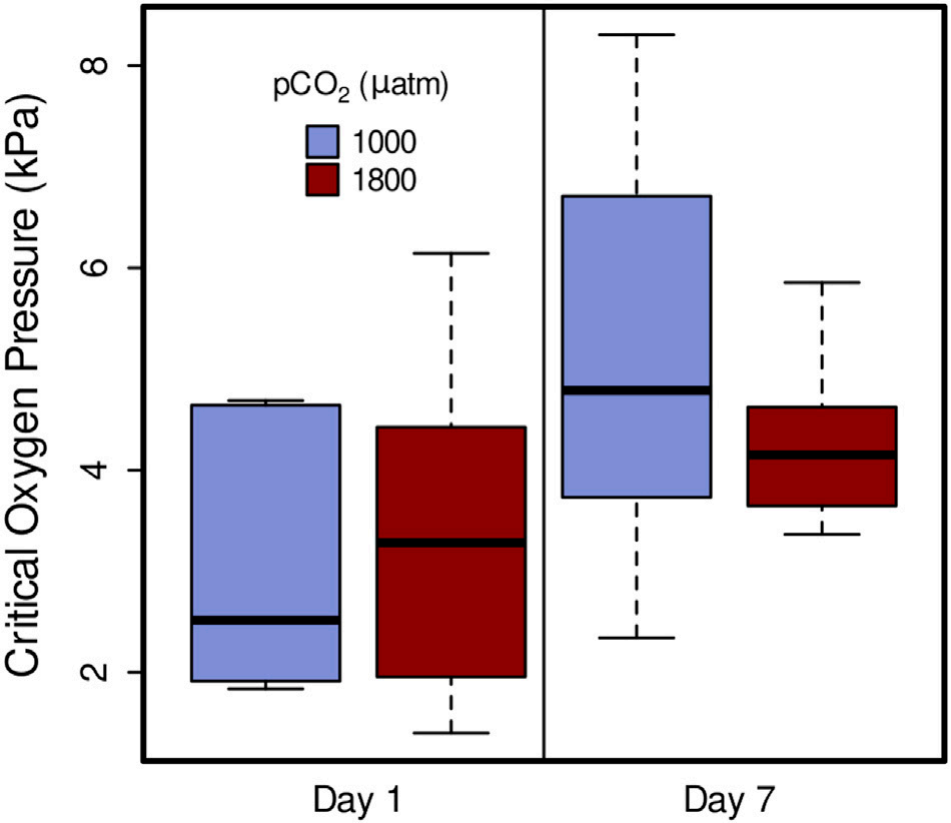
Critical oxygen pressure (*P*_*crit*_) of *Muusoctopus leioderma* after 1 day and 7 day exposure to carbon dioxide partial pressure (*p*CO_2_) of 1,000 µatm (purple, Day 1 *n* = 7, Day 7 *n* = 7) and 1800 µatm (maroon, Day 1 *n* = 8, Day 7 *n* = 7). Critical oxygen pressures are not significantly different between *p*CO_2_ treatments after long-term exposure, however, they did show an increase from day 1 and day 7 within both 1,000 µatm and 1800 µatm pCO_2_ treatments (linear mixed-effects model, x2 = 10.53, *p* = 0.001).

Critical oxygen partial pressure was not significantly affected by elevated *p*CO_2_, in 1 day treatments (1,000 µatm *P*_*crit*_ = 3.64 kPa, 1800 µatm *P*_*crit*_ = 3.14 kPa) or 7 day (1,000 µatm *P*_*crit*_ = 5.00, 1800 µatm *P*_*crit*_ = 4.50) (Figure 4; linear mixed-effects model, *x* ^*2*^ = 0.4646, *p* = 0.4954). However, it did show a significant increase from day 1 and day 7 within both 1,000 µatm and 1800 µatm *p*CO_2_ treatments (Figure 4; linear mixed-effects model, *x* ^*2*^ = 10.53*, p* = 0.001).

**FIGURE 5:**
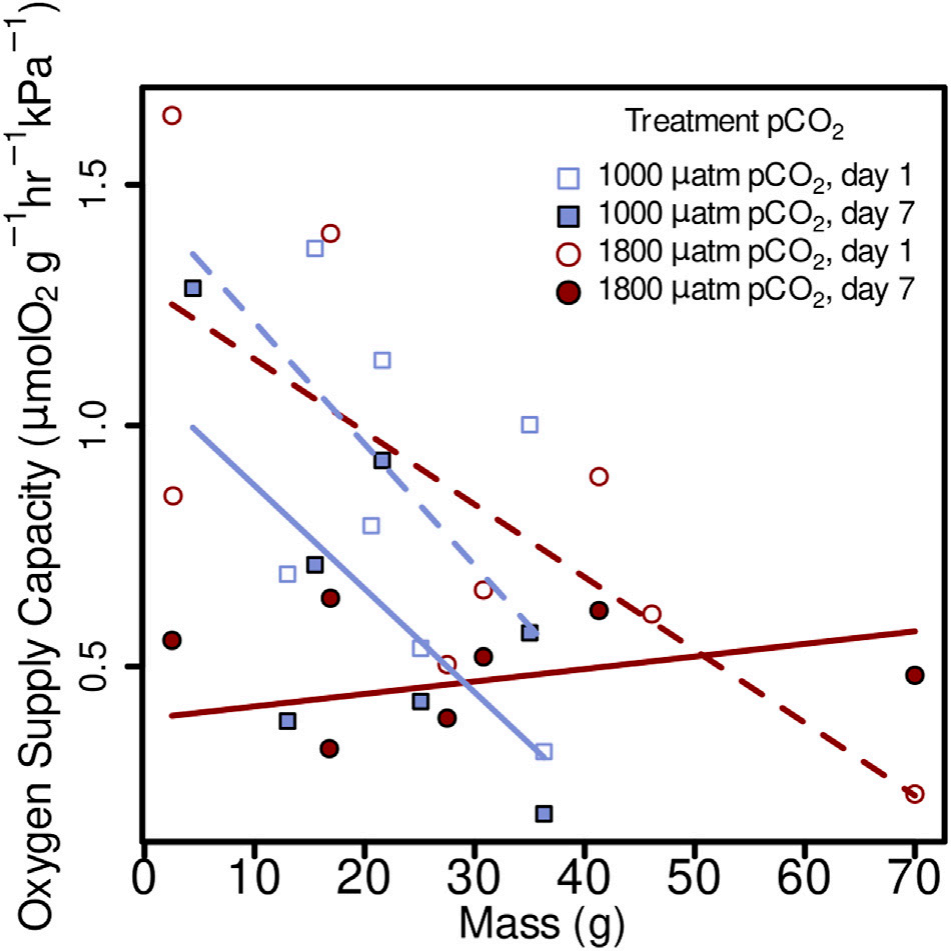
Oxygen supply capacity (α) from *M. leioderma* in Burrows Bay, Anacortes Washington held for 1 day (open symbols) and 7 days (closed symbols) at 1,000 µatm (purple squares) or 1,800 µatm (maroon circles) pCO_2_ expressed as a function of body mass (M). Oxygen supply capacity was significantly affected by body mass (M), decreasing α with increased body mass in three of four treatments as follows: 1,000 µatm 1 day α = 1.47 + (−0.025)M, 1,000 µatm 7 day α = 1.09 + (−0.021)M, 1800 µatm 1 day α = 1.29 + (−0.015)M, and 1800 7 day α = 0.39 + 0.003M.

Oxygen supply capacity was not significantly affected by elevated pCO_2_, in 1 day treatments (1,000 µatm α= 0.80 µmol O_2_ g^−1^ hr^−1^ kPa^−1^, 1800 µatm *α* = 0.89 µmol O_2_ g^−1^ hr^−1^ kPa^−1^) or 7 day (1,000 µatm *α* = 0.53 µmol O_2_ g^−1^ hr^−1^ kPa^−1^, 1800 µatm *α* = 0.46 µmol O_2_ g^−1^ hr^−1^ kPa^−1^) (Figure 5; linear mixed-effects model, x2 = 0.06, *p* = 0.8080). However, it did show a decrease from day 1 and day 7 within both 1,000 µatm and 1800 µatm pCO_2_ treatments (Figure 5; linear mixed-effects model, *x* ^*2*^ = 17.35, *p* = 0.000031). There was an effect of mass on oxygen supply capacity in all four treatments as follows: 1,000 µatm 1 day *α* = 1.47 + (−0.025M), 1,000 µatm 7 day *α* = 1.09 + (−0.021M), 1800 µatm 1 day *α* = 1.29 + (−0.015M), and 1800 7 day *α* = 0.39 + 0.003M (Figure 5; linear mixed-effects model, x2 = 9.84, *p* = 0.00171).

## Discussion

This study is the first to investigate the effects of near-future ocean acidification on the physiology of a deep-water octopus. The Salish Sea is an excellent location for studying the effects of ocean acidification. It has historically had a persistent elevated *p*CO_2_, attributed in part to upwelling from the California Undercurrent (Murray et al., 2015). It is common to see *p*CO_2_ above 1,200 µatm which exceeds all but the most extreme atmospheric *p*CO_2_ projections for the end of this century (Onthank et al., 2021). Organisms that live in the Salish sea are persistently exposed to elevated *p*CO_2_ throughout their life history, making them excellent study subjects to explore the impact OA may have on such organisms after many generations.

Previous work on cephalopods has shown conflicting outcomes, with squid having a decrease in RMR and increase in *P*_*crit*_ when exposed to *p*CO_2_ above 1,000 µatm (Rosa and Seibel, 2008; Rosa and Seibel, 2010; Hu et al., 2014; Seibel, 2016), as well as no effect (Birk et al., 2018). The observed negative effect on aerobic metabolism has been postulated to be the result of challenges to respiratory physiology. Cephalopods rely on hemocyanin as a respiratory pigment which has a pronounced Bohr effect, resulting in a decrease in oxygen affinity in areas of low pH. Because of this, it has previously been predicted that cephalopods would not tolerate large changes in environmental pH (Portner and Zielinski, 1998; Rosa and Seibel, 2008; Seibel, 2016). However recent work by Birk et al. (2018) suggests that epipelagic squid blood oxygen carrying capacity is minimally impacted by changes in environmental pH. Yet recent work in octopus suggests this may not be valid for all cephalopods. *Octopus rubescens* exposed to elevated *p*CO_2_ shows a short-term elevation in RMR, however there was no change in RMR after 5 weeks and a significant increase in *P*_*crit*_ at the same time point, indicating a reduction in oxygen supply capacity resulting in decreased hypoxia tolerance (Onthank et al., 2021). This deviation from the prediction of Birk and Seibel’s model may be linked to the difference in Bohr coefficient found in some species of octopus as compared to those found in squid. In their proposed model squid were assigned a Bohr coefficient of −1.5 as being a worst-case scenario for considering impacts of OA on cephalopods. However octopus hemocyanin has an even larger Bohr effect with Bohr coefficients of −1.7 in *Enteroctopus dofleini* (Miller, 1985) and −1.99 in *Octopus macropus* (Lykkeboe and Johansen, 1982). This suggests that elevated *p*CO_2_ may have a greater effect on octopus blood oxygen binding and aerobic metabolism.

Here we found, unlike the decreased RMR reported in squid (Rosa and Seibel, 2008) or the short term elevated RMR in response to hypercapnia observed in *Octopus rubescens* (Onthank et al., 2021), *Muusoctopus leioderma* RMR was not significantly impacted when exposed to 1800 µatm. Additionally there was no significant change in *α* or *P*_*crit*_ as a result of exposure to hypercapnia. This does not follow most of the trends observed in other octopus or squid species previously studied. Our results do however show a change in both *α* and *P*_*crit*_ in both CO_2_ treatments with day 7 *P*_*crit*_ values being higher than those recorded for 1 day (Figure 4). It could be that these changes are the result of repeated exposure to low oxygen in subsequent *P*_*crit*_ measurements, however, this would be contrary to previous work showing a decrease in *P_crit_* with repeated hypoxia exposure in octopus (Onthank, 2008). The combination of no change in RMR, indicating the animals are not significantly stressed, with changes to *α* and *P_crit_* are particularly puzzling. This octopus species spends a considerable proportion of time in subsurface sediment burrows, which, as discussed below, would likely expose them to regular bouts of hypoxia. This would likely drive increases in supply capacity, such as elevating the amount of respiratory pigments to meet metabolic demands. This hypothesis is supported by our finding the oxygen supply capacity in *M. leioderma* (α = 0.80 µmol O2 g-1 hr-1 kPa-1 under control conditions) is substantially higher than other shallow living octopus species for which *α* is known (*Octopus vulgaris*: 0.30 µmol O2 g-1 h^−1^ kPa^−1^, *Octopus bimaculoides*: 0.34 µmol O_2_ g^−1^ hr^−1^ kPa^−1^, *Octopus rubescens*: 0.32 µmol O_2_ g^−1^ hr^−1^ kPa^−1^, as well as the deep sea octopus *Octopus californicus*: 0.23 µmol O_2_ g^−1^ hr^−1^ kPa^−1^ (Table 3) (Seibel and Childress, 2000; Valverde and García, 2005; Seibel and Deutsch, 2020; Onthank et al., 2021). However, similar to how terrestrial organisms decrease hematocrit as they acclimate to elevated oxygen pressure at lower elevations (Keys et al., 1986; Zubieta-Calleja et al., 2007; Borras et al., 2010), it is possible that the introduction of these octopuses to the well-oxygenated tanks in the lab initiated acclimation processes which lower oxygen supply capacity, such as a reduction of blood hemocyanin concentration or a reduction in hemocyanin oxygen affinity, while not impacting RMR. Such a decrease in oxygen affinity of hemocyanin when exposed to elevated oxygen concentrations has been demonstrated in the shore crab *Carcinus maenas* (Lallier et al., 1987). Unlike those in other treatments, octopuses in 1800 µuatm pCO_2_ at 7 days showed a positive relationship between oxygen supply capacity and mass. The reasons for this are unclear, but the unusual responses at the extremes of octopus body masses may be driving this. The largest individual (70 g) in 1800 µatm pCO_2_ is the only one that showed an increase in oxygen supply capacity, while the smallest individual (2.5 g) in 1800 µatm pCO_2_ showed the largest decrease in oxygen supply capacity in the study. Combined, this suggests a mass specific and pCO_2_ specific response in alpha to time in treatment, but more data would be required to make a firm conclusion.

**TABLE 3:** Comparative routine metabolic rate (RMR), critical partial pressure (*P_crit_*) and oxygen supply capacity (*α*) for species of cephalopods from the literature compared to those recorded for *Muusoctopus leioderma* in the present study.

Species Name	RMR (µmol O_2_ g^−1^ hr^−1^)	P_crit_	α	Temperature (°C)	References
Octopuses					
*Muusoctopus leioderma*	2.6	3.64	0.80^a^	13–14	Present study
*Octopus californicus*	0.6		0.23^b^	6	Seibel and Childress, (2000)
*Octopus bimaculoides*	0.73	2.13	0.34^c^	10	Seibel and Childress (2000)
*Octopus rubescens*	1.49	4.71	0.32^c^	11	Onthank et al. (2021)
*Octopus vulgaris*	2.36	8	0.30^c^	25	Wells et al. (1983a), Wells et al. (1983b), Valverde and García (2005)
Other Cephalopods					
*Vampyroteuthis infernalis*	0.07	0.96	0.07^c^	5	Seibel et al. (1997)
*Dosidicus gigas*	5.91	1.6	3.69^c^	10	Trueblood and Seibel, (2013)
*Nautilus pompilius*	1.09	6.47	0.17^c^	21	O’dor et al. (1990), Staples et al. (2000)

Methods of calculating alpha.

^a^ Estimated marginal mean (co-variate corrected mean) from linear mixed effects model.

^b^ Calculated directly from data extracted from figure.

^c^ Estimated by dividing mean RMR, by mean P_crit_.

*Muusoctopus leioderma* from this study lives in close geographic proximity to *Octopus rubescens*, and as such they encounter similar abiotic environmental factors. It would be reasonable to assume then these two species should have a similar response to elevated *p*CO_2_, yet they do not. The observed variation in responses to elevated *p*CO_2_ may be linked to differences in phylogenies and behavior between the two species.

### Phylogenetic influence

*Muusoctopus leioderma* has historically been considered a meso to bathyal benthic species, residing between 200–1,500 m depth, with a greatest occurrence between 400–650 m (Conners et al., 2016). This species is part of the Family Enteroctopodidae which includes three genera and a total of ∼33 species (Ibáñez et al., 2016; Jereb et al., 2016; Sanchez et al., 2018). Of these, all are deep living species with very few exceptions such as most of the species of genus *Enteroctopus* which are found as deep as 1,500 m, but which can also be found in shallow water. There is one instance of *Muusoctopus eureka* found at 20 m (Laptikhovsky et al., 2011) and *Muusoctopus leioderma* at 70 m (Hochberg, 1998) and at 10 m in the present study. Otherwise, all other species occupy depth ranges between 200 and 1,500 m. Along the west coast of North America there is a pronounced oxygen minimum zone, marked by low oxygen and high levels of CO_2_ above 2000 µatm (Paulmier et al., 2011) that occurs between approximately 300–800 m (Kamykowski and Zentara, 1990). These depths overlap with the depth of greatest abundance of *M. leioderma* (Conners et al., 2016)*.* The population of *M. leioderma* used in this study is the shallowest occurrence recorded for any species in the genus *Muusoctopus* that we are aware of. Yet this population of *M. leioderma* presents classic deep sea features, having no ink sac, minimal chromatic change, large eyes, and can only be found out of its burrows in the dark. As a member of Family Enteroctopodidae, there is a long evolutionary history of deep occurrence. It is logical that as *M. leioderma* seemingly has adapted anatomy for the deep sea it’s physiology would have as well.

Conversely, *Octopus rubescens,* a member of family Octopodidae, is the most abundant shallow water octopus along the west coast of North America. Its distribution ranges from the mouth of the Gulf of California, Mexico to the Gulf of Alaska. It is common in the intertidal and has been found as deep as 300 m (Hochberg, 1998). These depths are shallower than the depths of OMZs along the west coast of North America typically occur (Kamykowski and Zentara, 1990). Like other shallow water species *O. rubescens* has an ink sac, performs dynamic skin color and texture change, (Packard and Hochberg, 1977), and is most active during the day (Humbert et al., 2022). This species and its shallow water congeners have not experienced the same environmental hypoxia and hypercapnia of species who live at depths corresponding to OMZs and thus may not have the same physiological adaptations.

The Salish Sea receives water input from the upwelling of the California Undercurrent (Murray et al., 2015). As the CU upwells there is additional consumption of oxygen and production of CO_2_ from respiration in shelf sediments (Connolly et al., 2010; Bianucci et al., 2018) resulting in *p*CO_2_ levels in excess of 2,100 µatm and oxygen levels below 30% air saturation (Murray et al., 2015). It is probable that the population from this study was originated by individuals brought into the Salish Sea from CU upwelling. The *P*_*crit*_ we recorded at 1,000 and 1800, (Figure 4), are close to *P*_*crit*_ values recorded for other species of cephalopods who regularly experience enter OMZs, such as *Dosidicus gigas* (Trueblood and Seibel, 2013). While oxygen supply capacity is substantially lower than *D. gigas*, a transient of the OMZ, it is higher than that of *Vampyroteuthis infernalis*, a resident of the OMZ, and more than double that of shallow living octopods as discussed above (Table 3). This supports that *M. leioderma* may have adapted its oxygen supply capacity for the hypoxic waters like those of the CU. Similarly, the lack of significant change in RMR, *P*_*crit*_ and *α* when exposed to 1800 µatm supports this population is adapted to life in environmental hypercapnia, similar to what is found in the CU. The response observed in *O. rubescens* by Onthank et al. (2021) at 1,500 µatm may reflect that this level of *p*CO_2_ is above historical levels this shallow living species has encountered, but is within the range it is able to acclimate to.

### Denning and burrowing behavior

*Octopus rubescens,* like other species of octopus, spends the majority of each 1 day period in a den (Kayes, 1973; Mather, 1988; Humbert et al., 2022). While their natural dens in the wild have not been studied extensively, they are regularly observed using rocky outcroppings, large empty barnacle shells, beer bottles, and other hard containers of sufficient size (Anderson, 1997; Anderson et al., 1999)*.* These containers allow for reasonable water exchange, and minimal background respiration besides that of the occupying octopus. As such, it is unlikely that *O. rubescens* would typically experience extreme hypoxia or hypercapnia while occupying its den.

*Muusoctopus leioderma* spend an appreciable amount of time in burrows as well. During this study we only observed them on silt bottoms at night, never during the day. Observations in the lab show their burrows to be small, not much larger than the volume of the individual’s body, and having a small narrow opening at least one mantle length below the surface of the substrate. The interior of silty coastal bottoms such as those found in Burrows Bay are often areas of low oxygen and elevated CO_2_ (Middelburg et al., 2005). By creating and occupying a small burrow and spending large portions of each day there, it is likely that *M. leioderma* regularly experiences hypoxia and hypercapnia and as a result has physiological adaptations for both.

### Invasion potential of deep-water fauna

In retrospect, it is unsurprising that our treatments resulted in no significant change in RMR or *P*_*crit*_ given the typical high *p*CO_2_ of most of *M. leioderma*’s depth range. Despite being first described in 1911 (Berry, 1911), and collected frequently as bycatch in various trawl fisheries (Conners et al., 2016), this species has only recently been found as shallow at 10 m depth.

Depth of occurrence, and the resulting zonation of species has been attributed to environmental factors such as temperature, oxygen, and/or food availability, which impact the physiology of organisms, and competition (Rex, 1976; Collins et al., 1999; Carney, 2005; Yeh and Drazen, 2009). Deep-water animals living in higher *p*CO_2_ environments will likely be more robust to higher *p*CO_2_ than their shallow water counterparts. As *p*CO_2_ continues to rise in shallow waters, this could potentially shift balances that may have historically excluded deep-water animals from shallow-water environments. If this is true, the first locations you would expect to see such shallow-water invasions would be in areas that already experience relatively high *p*CO_2_, and recent anthropogenic inputs pushing CO_2_ levels even higher, such as the Salish Sea (Murray et al., 2015; Onthank et al., 2021). In addition, the first animals you would likely see invading into shallow waters would be, like octopuses, highly mobile and highly adaptable. Together, the finding that an octopus most commonly found in 450–650 m at the unprecedented depth of 10 m of water is relatively robust to elevated *p*CO_2_ could both partially explain its recent discovery in shallow water and also be a harbinger of future deep-to-shallow water invasions.

## Conclusion

Our data is the first of its kind, examining the effects of OA on the aerobic metabolism of a bathyl species of octopus. We show that *Muusoctopus leioderma* maintained its RMR and *P*_*cri*t_ at elevated levels of 1800 µatm *p*CO_2_, even after 7 days of exposure. The ability to maintain aerobic physiology at these relatively high levels shows this species has physiological adaptations that are likely linked to its phylogeny and life history. It is unique to find this species in the shallow depths recorded in this study. However, it may be that their robust tolerance of elevated *p*CO_2_ has allowed them to survive in the hypercapnic shallow waters of the Salish Sea. Further research is needed to clarify the mechanism that drives this species tolerance to high environmental *p*CO_2_. Because of their resilience to acidification, and relative ease of collection and maintenance in the lab, *Muusoctopus leioderma* make an excellent model system to further study how some organisms may compensate for future levels of environmental *p*CO_2._

## Data Availability

The datasets presented in this study can be found in online repositories. The names of the repository/repositories and accession number(s) can be found below: https://doi.org/10.5281/zenodo.7058934 Zenodo.org.

## Supplementary material

Supplementary Figure 1 Flow chart of experimental procedures for examining the effect of elevated *p*CO_2_ on Muusoctopus leioderma from Burrows Bay, Washington United States.

Supplementary Figure 2 Diagram of flow through respirometry system used for measuring routine metabolic rate (RMR) and critical partial pressure (*P_crit_*). A firesting optode system (Pyroscience GmbH Aachen Germany) was used to measure oxygen content of water at the inlet and outlet of the respirometer. A peristaltic pump was used to pull water through the system, blue arrows denote direction of water flow. Flow rates for each respirometer were measured at the start and end of each respirometry run by measuring output of water in mL for 1 min. The respirometer system was submerged in a water bath to maintain temperature. To measure Pcrit the system was closed by connecting the inlet and outlet ports.
