# Supplementary figures and images for: Bathyal octopus, Muusoctopus leioderma, living in a world of acid: First recordings of routine metabolic rate and critical oxygen partial pressures of a deep water species under elevated pCO2
Source: Front Physiol. 2022 Dec 1;13:1039401. doi: 10.3389/fphys.2022.1039401 (PMC9751821; doi:10.3389/fphys.2022.1039401)

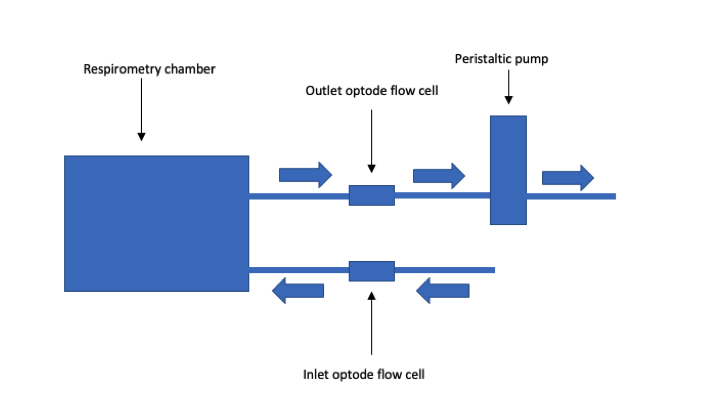

Supplement: Supplementary file 1 [file Image2.PNG]

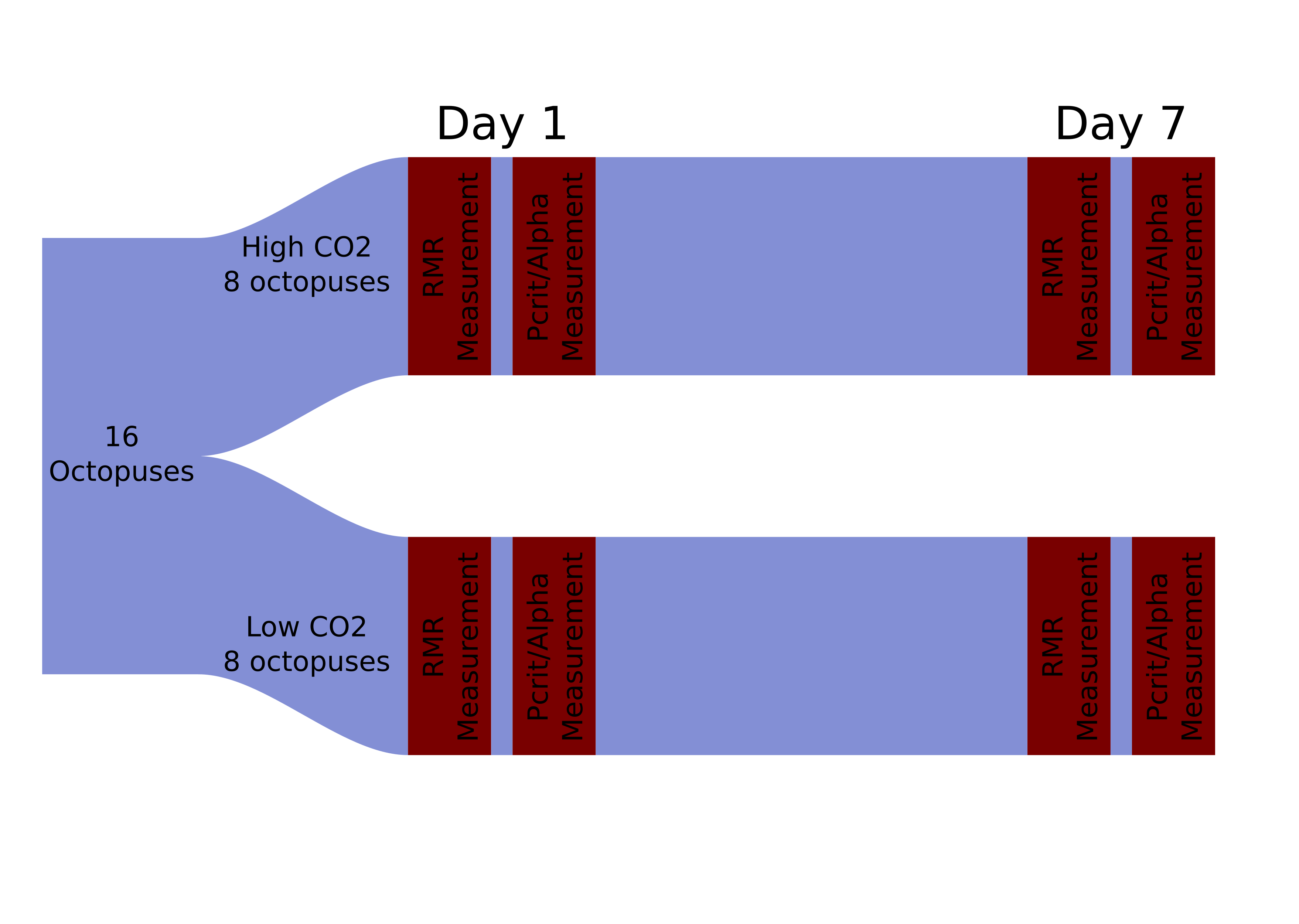

Supplement: Supplementary file 2 [file Image1.PNG]
